# Modeling and Vibration Analysis of Carbon Nanotubes as Nanomechanical Resonators for Force Sensing

**DOI:** 10.3390/mi15091134

**Published:** 2024-09-06

**Authors:** Jun Natsuki, Xiao-Wen Lei, Shihong Wu, Toshiaki Natsuki

**Affiliations:** 1 Institute for Fiber Engineering and Science (IFES), Interdisciplinary Cluster for Cutting Edge Research (ICCER), Shinshu University, Ueda 386-8567, Japan; jnatsu@shinshu-u.ac.jp; 2 School of Materials and Chemical Technology, Tokyo Institute of Technology, 2-12-1 Ookayama, Meguro-ku, Tokyo 152-8552, Japan; lei.x.ac@m.titech.ac.jp; 3 Precursory Research for Embryonic Science and Technology (PRESTO), Japan Science and Technology Agency (JST), Saitama 332-0012, Japan; 4 Department of Electrical and Electronic Engineering, College of Intelligent Science and Engineering, Yantai Nanshan University, Longkou 265713, China; 5 College of Textiles and Apparel, Quanzhou Normal University, Quanzhou 362000, China; 6 Faculty of Textile Science and Technology, Shinshu University, 3-15-1 Tokida, Ueda 386-8567, Japan

**Keywords:** carbon nanotubes, vibration frequency, nanosensor, modeling

## Abstract

Carbon nanotubes (CNTs) have attracted considerable attention as nanomechanical resonators because of their exceptional mechanical properties and nanoscale dimensions. In this study, a novel CNT-based probe is proposed as an efficient nanoforce sensing nanomaterial that detects external pressure. The CNT probe was designed to be fixed by clamping tunable outer CNTs. By using the mobile-supported outer CNT, the position of the partially clamped outer CNT can be controllably shifted, effectively tuning its resonant frequency. This study comprehensively investigates the modeling and vibration analysis of gigahertz frequencies with loaded CNTs used in sensing applications. The vibration frequency of a partially clamped CNT probe under axial loading was modeled using continuum mechanics, considering various parameters such as the clamping location, length, and boundary conditions. In addition, the interaction between external forces and CNT resonators was investigated to evaluate their sensitivity for force sensing. Our results provide valuable insights into the design and optimization of CNT-based nanomechanical resonators for high-performance force sensing applications.

## 1. Introduction

Carbon nanotubes (CNTs) have emerged as promising candidates for various nanotechnology applications due to their exceptional mechanical, electrical, and thermal properties [1,2,3,4]. Among these applications, CNT-based nanomechanical resonators have gained significant attention because of their potential for highly sensitive force sensing [5,6,7,8,9]. Such sensitive nanosensor materials are crucial for nanoscale force sensing in a wide range of fields, including nanoelectromechanical systems (NEMS), biological sensing, and environmental monitoring.

Previous studies have reported the use of CNTs as piezoresistive force sensors, which have demonstrated high sensitivity for detecting various forces and high efficiency as piezoresistors in sensor applications [10,11,12,13]. CNTs have many unique properties that are advantages for fabricating next-generation structural health monitoring systems. These films exhibit good sensing stability, linearity, sensitivity, and repeatability within a practical strain range [13]. Cullinan and Culpepper investigated the use of CNTs as piezoresistive micro-electromechanical sensors through both theoretical and experimental approaches [14]. They reported that CNT-based strain sensors could overcome certain limitations of existing small-scale force/displacement sensing technologies. A theoretical model and approach were developed to predict the gauge factor for general CNTs. The simulation results indicated that the performance of CNT-based piezoresistive sensor systems could be improved by an order of magnitude if the CNTs could be accurately sorted based on the chirality.

A particularly interesting application of CNTs is their potential as nanomechanical resonators for force sensing. The ability of CNTs to vibrate in response to external forces opens up exciting possibilities for the development of highly sensitive, miniaturized sensors capable of detecting minute changes in force with unprecedented precision. Recently, stretchable sensors that can be incorporated into clothing have attracted significant interest for applications in smart textiles and flexible electronics. Previous studies have successfully fabricated the stretchable and wearable piezoresistive sensors using high-performance CNT-based nanocomposites [15,16,17,18]. The appropriate CNT content increased both the sensitivity and stability of the fabricated sensor [15]. The electromechanical characteristics of the strain sensors were based on the intrinsic resistive responsive response behaviors of CNT-based nanocomposites [18]. The high relative change in resistance of the CNT-based nanocomposites under tensile strain can be attributed to the tunneling conductivity [19,20]. However, these studies primarily focused on using CNTs as additives in composite materials. Wang and Musameh reported the development of CNT-derived screen-printed electrochemical sensors using CNT ink [21]. Printing technologies facilitate sensing applications by enabling large-area, high-throughput production of electronics and sensors on mechanically flexible substrates [22]. Film materials based on the CNT network demonstrated significant deformation resistance, making them excellent candidates for use as electrical interconnects and electrodes. Fan et al. [23] modeled and simulated an ultrasensitive position/force nanotransducer based on a nanogap-involved concentric nanostructure. Their theoretical study of the nanowire architecture, along with the characterization techniques used, provided valuable insights for implementing such nanowire-based sensors.

Advances in nanotechnology have enabled the development of various structures, including the precise control of the diameter and length of CNTs [24,25,26,27] as well as the positions of telescoping CNTs [28,29]. The controlled and reversible telescopic extension of CNTs was fabricated by Cumings and Zettl [29]. The repeated extension and retraction of the stretchable nanotube segments showed minimal wear or fatigue at atomic scale, resulting in a nearly perfect wear-free surface for telescopic multiwalled-CNTs. Based on the novel telescopic CNT structures, the retraction energy of CNTs, driven by an extruded core through van der Waals (vdW) force, induces core oscillations, creating nanomechanical systems capable of operating at frequencies beyond several gigahertz [29,30,31]. The telescoped length of a double-walled CNT can be precisely controlled to match its resonant frequency with a signal frequency, allowing the telescoped CNT to be tuned for use as a bandpass component [29]. These studies have demonstrated that resonance frequencies can be effectively altered by controllably sliding the core-nanotube within its outer nanotube casing.

NEMS based on telescoped CNTS has attracted significant attention from the research community for various applications. By designing the resonant frequency of the CNT oscillator as a controllable parameter via a telescopic outer tube, advanced functionality can be achieved for their use as NEMS components [32]. Telescoping multi-walled CNTs (MWCNTs) are particularly suitable for use as atomic force microscopy (AFM) probes, as they are sufficiently long and thin to capture detailed images of sample surfaces with deep holes [33,34]. Jensen et al. demonstrated a tunable nanoscale mechanical resonator with a broader frequency range than competing designs, demonstrating potential applications in precise mass and force measurements [35].

Since CNTs are very sensitive to small applied stresses and strains [36,37], single CNTs can be proposed as resonators for nanoscale force measurements. The sensing principle is based on the resonant frequency shift of CNT resonators when subjected to an externally applied load. Notably, the resolution of a CNT resonator is influenced by the size and structure of the CNT. In this study, we present a novel tunable oscillator model using a telescoping double-walled CNT probe as a force sensor. The frequency tuning function of the CNT probe is achieved by adjusting the position of the outer nanotubes relative to the fixed core nanotube, with the core and outer nanotubes coupled through van der Waals (vdW) interactions. An analytical procedure based on continuum mechanics was developed to investigate the vibration frequency of the CNT probe. Classical continuum mechanics theory typically considers the continuum limit of lattice models for nanostructures. In this study, nonlocal continuum mechanics is introduced for nanostructured materials to account for small-scale effects, providing a reliable method for modeling nanoresonators. By advancing our understanding of CNT-based nanomechanical resonators, this study contributes to the development of next-generation nanoforce sensing technologies.

## 2. Modeling Procedures

### 2.1. Nonlocal Continuum Beam Model

In classical continuum elasticity, the stress state at a point is related only to the strain state at the given point. Eringen’s nonlocal elasticity theory [38,39,40] states that the stress at a point in an elastic continuum depends not only on the strain at that point but also on those at all points in the domain. The nonlocal continuum mechanics model allows the consideration of the small-scale effect that becomes significant when dealing with micro- or nanostructures with discrete domains.

Based on the Bernoulli-Euler beam theory combined with the nonlocal elasticity theory, the relationship between stress σ and strain ε can be expressed as [41]:

(1) $$\sigma -\mu^{2}\frac{\partial^{2}\sigma }{\partial x^{2}}=E\varepsilon$$

where E represents the elastic modulus, and *x* represents the axial coordinate. $\mu ={\left(e_{0}a\right)}^{2}$ is the nonlocal coefficient, where a denotes the internal characteristic length (e.g., length of the *C-C* bond for the CNT), and $e_{0}$ denotes a constant appropriate to each material.

For the transversely vibrating beam, the equilibrium equations can be written as follows [42,43]:

(2) $$V-\frac{\partial M}{\partial x}+P\frac{\partial w}{\partial x}=0$$

(3) $$\frac{\partial V}{\partial x}=-p+\rho A\frac{\partial^{2}w}{\partial t^{2}}$$

where *w* is the transverse displacement and *t* is the time variable. *V* is the shear force, *M* is the bending moment, *P* is the axial compressive load, and p is the uniformly distributed transverse load per unit axial length. ρ and A are the mass density and cross-sectional area of the beam, respectively.

According to Equation (1) and using the relation between strain and curvature $\varepsilon =-y\frac{\partial^{2}w}{\partial x^{2}}$, and the bending moment M=∫yσdA, the constitutive relation taking into the nonlocal influence can be given by

(4) $$M-\mu^{2}\frac{\partial^{2}M}{\partial x^{2}}=-EI\frac{\partial^{2}w}{\partial x^{2}}$$

where *I* is the moment of inertia, thus, EI denotes the bending stiffness of the beam.

Furthermore, by differentiating both sides of Equation (4) with respect to the variable *x* twice and using Equations (2) and (3), the governing differential equations of a beam under an axial compressive load *P* are obtained by

(5) $$EI\frac{\partial^{4}w}{\partial x^{4}}+\rho A\frac{\partial^{2}w}{\partial t^{2}}+P\frac{\partial^{2}w}{\partial x^{2}}-\mu^{2}\left(\rho A\frac{\partial^{4}w}{\partial x^{2}\partial t^{2}}+P\frac{\partial^{4}w}{\partial x^{4}}\right)=p-\mu^{2}\frac{\partial^{2}p}{\partial x^{2}}$$

and, the shear force and the bending moment are expressed as follows:

(6) $$V=-EI\frac{\partial^{3}w}{\partial x^{3}}+\mu^{2}\rho A\frac{\partial^{3}w}{\partial x\partial t^{2}}+\mu^{2}\left(P\frac{\partial^{3}w}{\partial x^{3}}-\frac{\partial p}{\partial x}\right)-P\frac{\partial w}{\partial x}$$

(7) $$M=-EI\frac{\partial^{2}w}{\partial x^{2}}+\mu^{2}\rho A\frac{\partial^{2}w}{\partial t^{2}}+\mu^{2}\left(P\frac{\partial^{2}w}{\partial x^{2}}-p\right)$$

**Figure 1:**
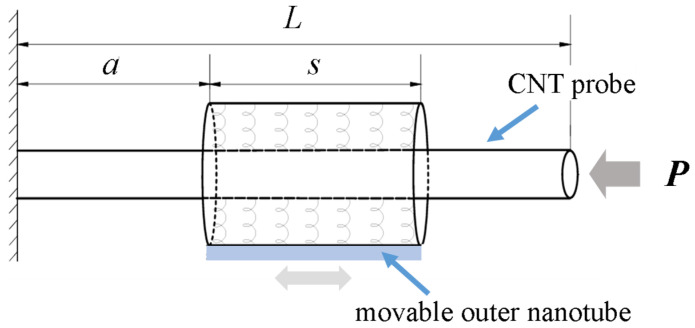
Analytical model of cantilevered CNT-based probe clamped in movable outer nanotube.

The proposed CNT-based probe was clamped in a movable outer nanotube and modeled, as shown in Figure 1. In the figure, *L* represents the length of the CNT probe, which is clamped by a tunable outer nanotube with the length *s*. The distance *a* indicates the position of the movable outer nanotube away from the fixed end. The movable outer nanotube can easily change the support position of the CNT probe. *P* represents the axial load acting on the CNT probe.

The interaction force $\left(p\right)$ in Equations (5) and (7) between the CNT probe and the supporting outer nanotube can be described as a Whitney–Riley model characterized by the vdW interaction coefficient $\left(k_{w}\right)$ per unit length and is given by

(8) $$p=-k_{w}w$$

and [44,45]

(9) $$k_{w}=\frac{320\times \left(2R\right)\;erg/{cm}^{2}}{0.16\;d^{2}},\;d=0.142\;nm$$

where R represents the nanotube radius.

Based on the Euler-Bernulli beam equation obtained in Equation (5), the governing differential equation of the CNT probe model shown in Figure 1 is given and divided into the following three parts:

**Nanotubes 1 and 3** $\left(p=0\right)$

(10) $$EI\frac{\partial^{4}w_{j}}{\partial x^{4}}+\rho A\frac{\partial^{2}w_{j}}{\partial t^{2}}+P\frac{\partial^{2}w_{j}}{\partial x^{2}}-\mu^{2}\left(\rho A\frac{\partial^{4}w_{j}}{\partial x^{2}\partial t^{2}}+P\frac{\partial^{4}w_{j}}{\partial x^{4}}\right)=0\;\;\;\;\;\;\;\;\;\;\;\left(j=1,\;3\right)$$

**Inner nanotubes 2** $\left(p=-k_{w}w\right)$

(11) $$EI\frac{\partial^{4}w_{2}}{\partial x^{4}}+\rho A\frac{\partial^{2}w_{2}}{\partial t^{2}}+P\frac{\partial^{2}w_{2}}{\partial x^{2}}-\mu^{2}\left(\rho A\frac{\partial^{4}w_{2}}{\partial x^{2}\partial t^{2}}+P\frac{\partial^{4}w_{2}}{\partial x^{4}}\right)=-k_{w}w_{2}+\mu^{2}k_{w}\frac{\partial^{2}w_{2}}{\partial x^{2}}$$

where subscripts 1 and 3 indicate the exposed parts of the CNT probe, and subscript 2 denotes the clamped part in the middle.

### 2.2. Solution of Governing Equations

In this study, because we address free vibrations, the vibrational solution of the differential equations can be described as follows:

(12) $$w_{j}=Y_{j}\left(x\right)e^{i\omega t},\;\;\;\;j=1,\;2,\;3$$

where ω denotes the vibrational frequency of the CNT probe, and $Y_{j}\left(x\right)\;\left(j=1,\;2,\;3\right)$ denote the vibration amplitudes for the exposed parts and the clamped part in the middle.

Substituting Equation (12) into Equations (10) and (11), the governing equations of the vibration property for the CNT probe are obtained as follows:

**Nanotubes 1** $\left(0\leq x<a\right)$ **and 3**  $\left(a+s\leq x<L\right)$

(13) $$\frac{d^{4}Y_{j}}{dx^{4}}+2\alpha \frac{d^{2}Y_{j}}{dx^{2}}-\beta Y_{j}=0,\;\;j=1,\;3$$

where

(14) $$2\alpha =\frac{P+\rho A\mu^{2}\omega^{2}}{EI-\mu^{2}P},\;\;\;\;\;\;\;\;\;\;\;\;\;\beta =\frac{\rho A\omega^{2}}{EI-\mu^{2}P}$$

The governing equation of motion of Equation (13) is a fourth-order differential equation, whose solutions can be given by characteristic roots.

(1) If $EI>\mu^{2}P$, we obtain
  (15) $$\alpha >0,\;\beta >0\;\mathrm{and}\;\alpha^{2}+\beta =\frac{{\left(P-\rho A\mu^{2}\omega^{2}\right)}^{2}+4EI\rho A\omega^{2}}{4{\left(EI-\mu^{2}P\right)}^{2}}>0$$

Thus,

(16) $$Y_{j}\left(x\right)=A_{j1}\cosh\gamma x+A_{j2}\sinh\gamma x+A_{j3}\cos\lambda x+A_{j4}\sin\lambda x\;j=1,\;3$$

where

(17) $$\gamma =\sqrt{-\alpha +\sqrt{\alpha^{2}+\beta }},\;\lambda =\sqrt{\alpha +\sqrt{\alpha^{2}+\beta }}$$

(2) If $EI<\mu^{2}P$

We obtain α, β<0 from Equation (14). Thus, the characteristic equation roots exhibit two groups of real numbers, which are given by

(18) $$\gamma =\sqrt{-\alpha +\sqrt{\alpha^{2}+\beta }},\;\mathrm{and}\;\lambda =\sqrt{-\alpha -\sqrt{\alpha^{2}+\beta }}$$

The solution of the differential Equation (13) can be expressed as

(19) $$Y_{j}\left(x\right)=A_{j1}\cos h\gamma x+A_{j2}\sin h\gamma x+A_{j3}\cos h\lambda x+A_{j4}\sin h\lambda x,\;\;j=1,\;3$$

**Inner nanotubes 2** $\left(a\leq x\ll a+s\right)$

(20) $$\frac{d^{4}Y_{2}}{dx^{4}}+p\frac{d^{2}Y_{2}}{dx^{2}}+q=0$$

where

(21) $$p=\frac{P+\rho A\mu^{2}\omega^{2}-\mu^{2}k_{w}}{EI-\mu^{2}P},\;\;\;q=\frac{k_{w}-\rho A\omega^{2}}{EI-\mu^{2}P}$$

The solutions of the governing equation for Equation (20) can be obtained by considering characteristic roots as follows:

(22) $$Y_{2}\left(x\right)=A_{21}e^{fx}+A_{22}e^{gx}+e^{kx}\left(A_{23}\cos lx+A_{24}\sin lx\right)$$

where f and g denote two real solutions of characteristic equation roots, and k and l denote the real and imaginary parts of complex conjugate k±jl, respectively.

If the solution of the characteristic equation is two groups of complex conjugates of f±jg and k±jl, we obtain

(23) $$Y_{2}\left(x\right)=e^{fx}\left(A_{21}\cos gx+A_{22}\sin gx\right)+e^{kx}\left(A_{23}\cos lx+A_{24}\sin lx\right)$$

When the characteristic equation roots are real numbers, the solutions of the governing equation can be given by

(24) $$Y_{2}\left(x\right)=A_{21}e^{fx}+A_{22}e^{gx}+A_{23}e^{kx}+A_{24}e^{lx}$$

For nanoresonators based on the movable support position of a cantilevered CNT probe, the corresponding boundary conditions are given as follows:

(1) for a fixed left end $\left(x=0\right)$
  (25) $$w_{1}\left(0\right)={\left.\frac{\partial w_{1}}{\partial x}\right|}_{x=0}=0;$$
(2) for a free right end $\left(x=L\right)$
  (26) $$-EI{\left.\frac{\partial^{2}w_{3}}{\partial x^{2}}\right|}_{x=L}+{\left.\mu^{2}\left(\rho A\frac{\partial^{2}w_{3}}{\partial t^{2}}+P\frac{\partial^{2}w_{3}}{\partial x^{2}}\right)\right|}_{x=L}=0,$$
  (27) $$-EI{\left.\frac{\partial^{3}w_{3}}{\partial x^{3}}\right|}_{x=L}+{\left.\mu^{2}\left(\rho A\frac{\partial^{3}w_{3}}{\partial x\partial t^{2}}+P\frac{\partial^{3}w_{3}}{\partial x^{3}}\right)\right|}_{x=L}-{\left.P\frac{\partial w_{3}}{\partial x}\right|}_{x=L}=0;$$
(3) for continuous conditions at the supporting position $\left(x=a\right)$,
  (28) $$w_{1}\left(a\right)=w_{2}\left(a\right),\;{\left.\frac{\partial w_{1}}{\partial x}\right|}_{x=a}={\left.\frac{\partial w_{2}}{\partial x}\right|}_{x=a},$$
  (29) $$-EI{\left.\frac{\partial^{2}w_{1}}{\partial x^{2}}\right|}_{x=a}+\mu^{2}{\left.\left(\rho A\frac{\partial^{2}w_{1}}{\partial t^{2}}+P\frac{\partial^{2}w_{1}}{\partial x^{2}}\right)\right|}_{x=a}=-EI{\left.\frac{\partial^{2}w_{2}}{\partial x^{2}}\right|}_{x=a}+\mu^{2}{\left.\left(\rho A\frac{\partial^{2}w_{2}}{\partial t^{2}}+P\frac{\partial^{2}w_{2}}{\partial x^{2}}\right)\right|}_{x=a}+\mu^{2}{k_{w}w}_{2}\left(a\right),$$
  (30) $$-EI{\left.\frac{\partial^{3}w_{1}}{\partial x^{3}}\right|}_{x=a}+\mu^{2}{\left.\left(\rho A\frac{\partial^{3}w_{1}}{\partial x\partial t^{2}}+P\frac{\partial^{3}w_{1}}{\partial x^{3}}\right)\right|}_{x=a}-P{\left.\frac{\partial w_{1}}{\partial x}\right|}_{x=a}=-EI{\left.\frac{\partial^{3}w_{2}}{\partial x^{3}}\right|}_{x=a}+\mu^{2}{\left.\left(\rho A\frac{\partial^{3}w_{2}}{\partial x\partial t^{2}}+N\frac{\partial^{3}w_{2}}{\partial x^{3}}\right)\right|}_{x=a}-P{\left.\frac{\partial w_{2}}{\partial x}\right|}_{x=a}+\mu^{2}k_{w}{\left.\frac{\partial w_{2}}{\partial x}\right|}_{x=a};$$
(4) for continuous conditions at the supporting position $\left(x=a+s\right)$,
  (31) $$w_{2}\left(a+s\right)=w_{3}\left(a+s\right),\;{\left.\frac{\partial w_{2}}{\partial x}\right|}_{x=a+s}={\left.\frac{\partial w_{3}}{\partial x}\right|}_{x=a+s},$$
  (32) $$-EI{\left.\frac{\partial^{2}w_{2}}{\partial x^{2}}\right|}_{x=a+s}+\mu^{2}{\left.\left(\rho A\frac{\partial^{2}w_{2}}{\partial t^{2}}+P\frac{\partial^{2}w_{2}}{\partial x^{2}}\right)\right|}_{x=a+s}+\mu^{2}{k_{w}w}_{2}\left(a+s\right)=-EI{\left.\frac{\partial^{2}w_{3}}{\partial x^{2}}\right|}_{x=a+s}+\mu^{2}{\left.\left(\rho A\frac{\partial^{2}w_{3}}{\partial t^{2}}+P\frac{\partial^{2}w_{3}}{\partial x^{2}}\right)\right|}_{x=a+s},$$
  (33) $$-EI{\left.\frac{\partial^{3}w_{2}}{\partial x^{3}}\right|}_{x=a+s}+\mu^{2}{\left.\left(\rho A\frac{\partial^{3}w_{2}}{\partial x\partial t^{2}}+P\frac{\partial^{3}w_{2}}{\partial x^{3}}\right)\right|}_{x=a+s}-P{\left.\frac{\partial w_{2}}{\partial x}\right|}_{x=a+s}+\mu^{2}k_{w}{\left.\frac{\partial w_{2}}{\partial x}\right|}_{x=a+s}=-EI{\left.\frac{\partial^{3}w_{3}}{\partial x^{3}}\right|}_{x=a+s}+\mu^{2}{\left.\left(\rho A\frac{\partial^{3}w_{3}}{\partial x\partial t^{2}}+N\frac{\partial^{3}w_{3}}{\partial x^{3}}\right)\right|}_{x=a+s}-P{\left.\frac{\partial w_{3}}{\partial x}\right|}_{x=a+s}.$$

By substituting the transverse deflections of the clamped and exposed parts of the CNT probe into the above boundary conditions of Equations (25) and (33), the simultaneous equation can be written in matrix form as follows:

(34) $${\left[\Omega \right]}_{12\times 12}{\left\{C\right\}}_{12\times 1}={\left\{0\right\}}_{12\times 1}$$

where the matrix ${\left[\Omega \right]}_{12\times 12}$ concludes the various parameters, such as the axial load acting on the CNT probe and the vibrational frequency, the movable outer nanotube length *s*, and the distance *a* that represents the position; ${\left\{C\right\}}_{12\times 1}$ are unknown integration constants. The variation solution of the vibrational frequency of the CNT probe under the axial compressive load can be obtained from a nontrivial solution of Equation (34).

## 3. Simulation Results and Discussion

The numerical results for the vibration of the CNT element subjected to axial load are presented based on the nonlocal elasticity theory. The CNTs as nanomechanical resonators had the following material constants and parameters: the elastic modulus and mass density of the CNT were 3.3 TPa and 2.3 g/cm^3^, respectively, the diameter of the CNT probe was 3.0 nm; the CNT length was 80 nm; and the effective thickness of the CNT was taken to be that of a graphite sheet (0.34 nm) [46,47,48].

**Figure 2:**
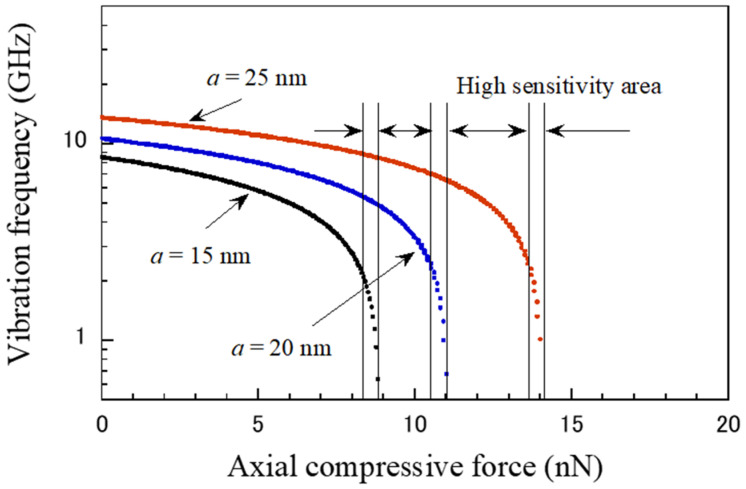
Dependence of vibrational frequency on external force in the CNT probe with clamping length (*s* = 20 nm) and different positions (*a*).

Various parameters were considered to investigate the effects of the applied force on the vibration frequency of the CNT probe. Figure 2 illustrates the relationship between the vibration frequency and the axial force applied to the CNT probe. The vibration frequency decreases rapidly with increasing axial force, which is affected by the supporting position. The CNT probe exhibits high sensitivity in force sensing and can detect nanoforces as small as less than 20 nN, as evidenced by the observed large frequency shifts. In this graph, where the x-axis represents force, the changes in vibrational frequency for forces below 20 nN clearly illustrate the probe’s sensitivity. The experimental and theoretical analysis results of previous studies [49,50,51] report that the buckling load of CNTs under compression is significantly larger than 20 nN, indicating that CNTs can serve as reliable mechanical sensing nanomaterials. In addition, the measurement sensitivity for a certain force range can be enhanced by adjusting the support position. For example, when the position *a* was changed from 25 nm to 15 nm, the sensitivity increased from 1.25 GHz/nN to 3.1 GHz/nN, a 1.5-fold increase. Note that there are critical compressive loads, e.g., 9, 11 and 14 nN, for different support positions. This means that the proposed CNT probe can be designed to obtain a more sensitive mechanical sensor that can measure a wider range of forces.

**Figure 3:**
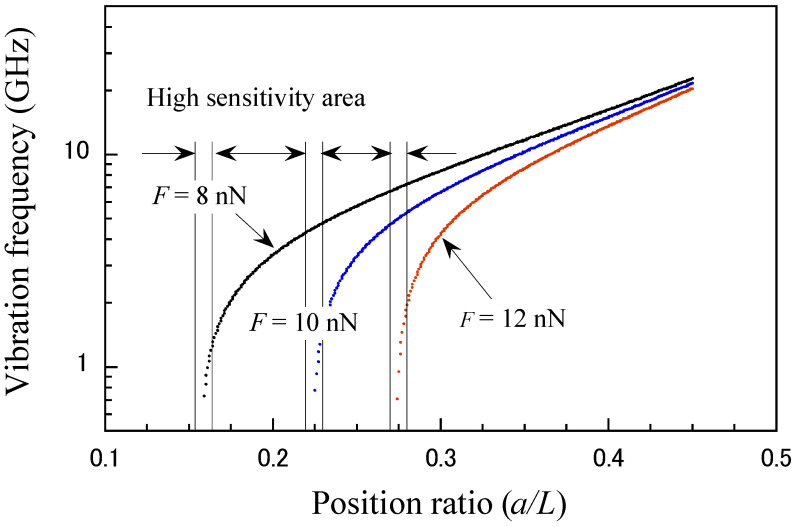
Dependence of clamping location on vibrational frequency in the CNT probe under different external forces *F* (*s* = 20 nm).

**Figure 4:**
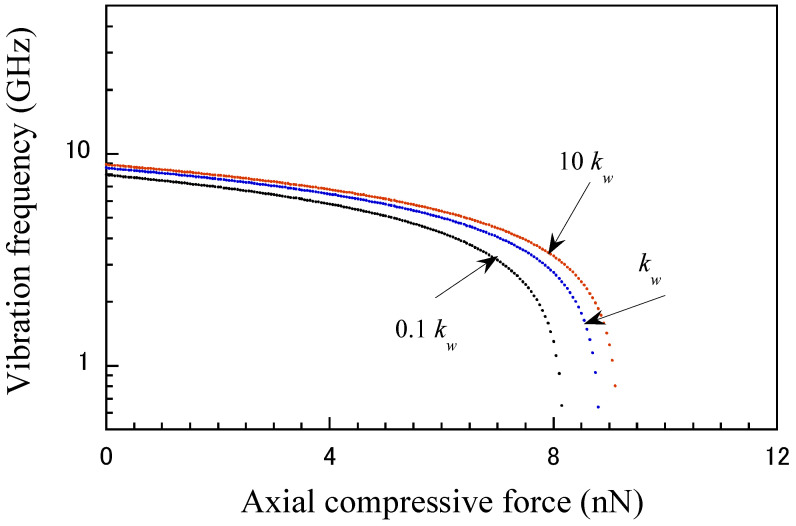
Influence of vdW interaction coefficient (*k_w_*) on the vibrational frequency of CNT at clamping position of *a* = 15 nm, and clamping length of *s* = 20 nm.

The variations in the vibration frequency are shown in Figure 3 as a function of the position ratio $\left(a/L\right)$. It is seen that the vibration frequency of the CNT probe decreases as the position ratio decreases. The influence of vibration frequency on the clamping position is more sensitive to high-level compressive load than to low-level load. This is because a higher axial force results in a high vibration frequency of the CNT probe. Figure 4 illustrates the influence of the vdW interaction coefficient (*k_w_*) on the vibration frequency of the CNT probe. These results suggest that the dependence of the intertube (vdW) interaction increases with increasing vibration frequency and becomes particularly significant near the critical compressive load.

**Figure 5:**
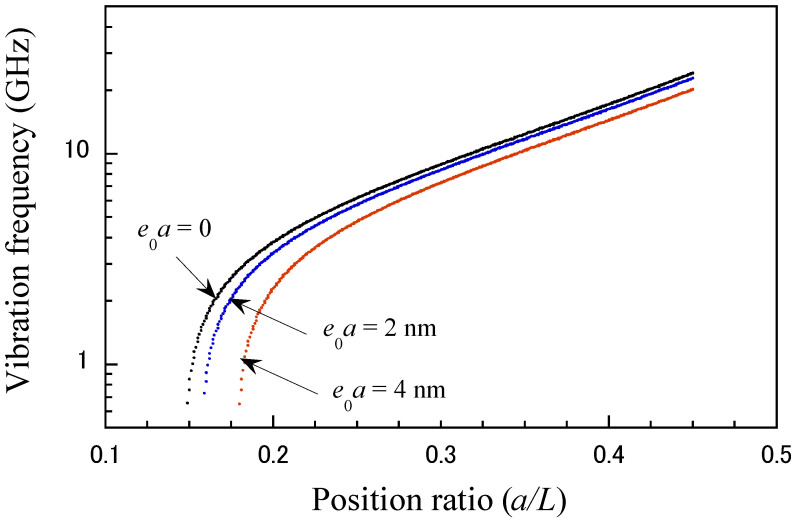
Relationship between the vibrational frequency and the position ratio for different nonlocal scale parameters (*s* = 20 nm, and an axial force of 8 nN).

**Figure 6:**
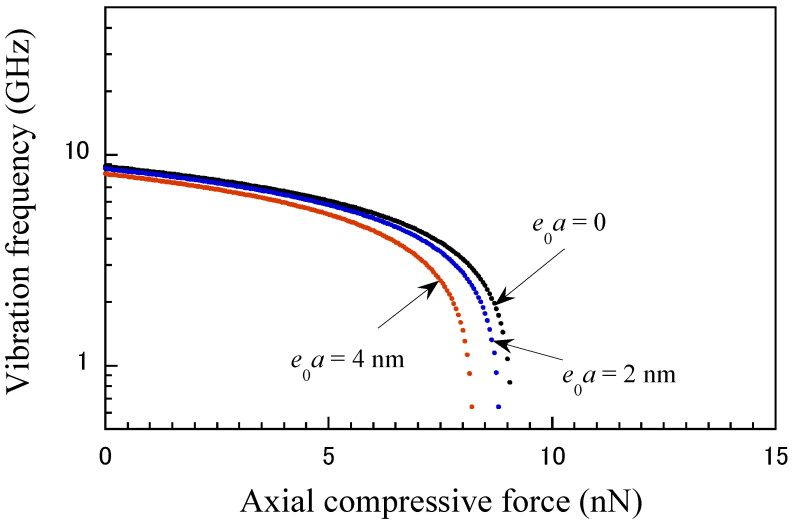
Relationship between vibrational frequency and axial compressive axial force for different nonlocal scale parameters (*a* = 15 nm, *s* = 20 nm).

In this study, the nonlocal elasticity theory was used to account for the size effect of discretely structured nanomaterials to achieve a more accurate vibration frequency response of the CNT probe. The nonlocal parameter $\left(\mu \right)$ was set to 0, 2.0, and 4.0 nm, which are generally suitable for the analysis of CNT beams [52,53,54]. Note that the nonlocal coefficient, μ=0, corresponds to the local elasticity theory. According to our simulation, the effects of the nonlocal coefficient on the vibration frequency are shown in Figure 5 and Figure 6 as functions of the support position and axial load, respectively. The vibration frequency of the CNT probe decreases as the nonlocal parameter increases, which is primarily due to the stiffness-softening effect caused by the nonlocal interactions. These interactions reduce the effective stiffness of the CNT probe, resulting in a lower resonance frequency. The nonlocal parameter plays a significant role in influencing the vibration frequency, which is especially noticeable at short position ratios (as shown in Figure 5) and under critical loads (as shown in Figure 6). This reduction in stiffness occurs because nonlocal interactions, such as long-range atomic or molecular forces, reduce the material’s resistance to deformation, thereby lowering the vibrational frequency of the CNT probe.

This suggests that considering the nonlocal effect is crucial for accurately predicting the vibration behavior of CNT probes, especially under certain conditions such as shorter support positions or near critical loads.

**Figure 7:**
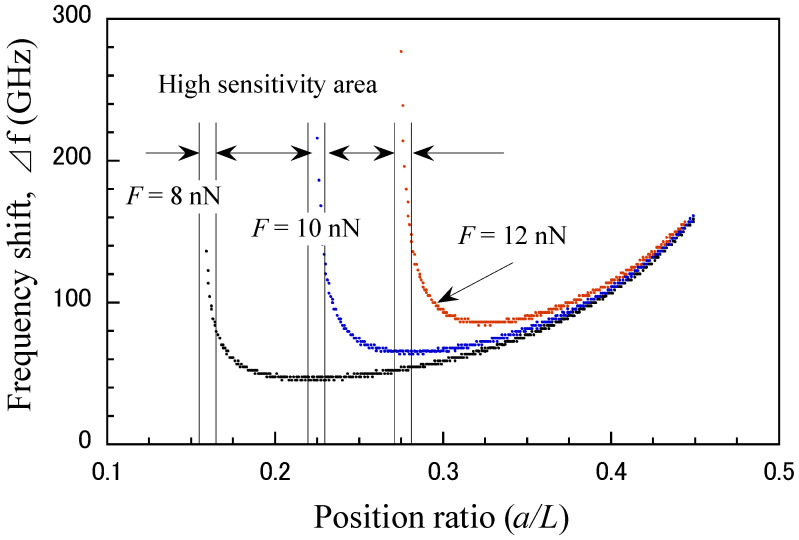
Dependence of the position ratio on the frequency shift under different external forces *F* (*s* = 20 nm).

Figure 7 shows the relationship between the frequency shift and variations in the support position under different external forces *F*. The frequency shift ∆f is defined as the change in the frequency due to the position ratio a/L. The force sensing sensitivity of the CNT probe varies significantly with the support position (a/L) under different applied loads. An external load applied to a CNT mechanical resonator deforms the CNT structure, changing its stiffness and resulting in a shift in the resonant frequency. In general, increasing the external load reduces the effective stiffness, resulting in a decrease in resonant frequency. This suggests that sensing performance can be enhanced by adjusting the position of the movable support outer nanotubes. For example, when 8 nN is applied, high sensitivity can be achieved by shifting a/L by about 0.15. Therefore, it is important to establish an ideal analytical model and rationally design the structures and sizes of CNT-based mechanical resonators. This sensitivity varies significantly with different applied loads, suggesting that precise control and optimization of the support position are critical for accurate force detection using the CNT probe.

## 4. Conclusions

We proposed a novel model of a double-walled CNT probe as a resonator for nanoscale force measurements, where the position of the clamped outer nanotubes can be adjusted. The force nanosensor was modeled and simulated based on the telescopic structure of the double-walled CNT probe. The interaction between the core and outer nanotubes was coupled using van der Waals (vdW) forces through simulation. The governing equations for the CNT probe model were constructed, and their solutions for the vibration frequency were obtained using nonlocal elasticity theory. Simulation results indicate that the vibration frequency of the proposed CNT probe is high, reaching the gigahertz range. The high resonant frequencies observed in the simulation are due to the intrinsic properties of carbon nanotubes (CNTs), such as their high stiffness and nanoscale size. These characteristics contribute to a higher natural frequency compared to conventional materials. The result shows that the sensitivity increased from 1.25 GHz/nN to 3.1 GHz/nN, which is a 1.5-fold increase when the position *a* was changed from 25 nm to 15 nm.

Additionally, the analysis reveals the sensitivity of CNT resonators to external forces, as manifested by shifts in resonant frequencies. This sensitivity enables the high-precision detection and quantification of applied forces, demonstrating the potential of CNT-based sensors in force sensing applications. The theoretical investigation and simulation results can serve as a reference for designing and developing ultrasensitive nanosensors and as a useful reference for further research on the mechanics of nanostructured materials.

## Data Availability

The original contributions presented in the study are included in the article, further inquiries can be directed to the corresponding author.

## References

[1] Ruoff R.S., Qian D., Liu W.K. (2003). Mechanical properties of carbon nanotubes: Theoretical predictions and experimental measurements. C. R. Phys..

[2] Qian D., Wagner G.J., Liu W.K., Yu M.F., Ruoff R.S. (2002). Mechanics of carbon nanotubes. Appl. Mech. Rev..

[3] Gardea F., Lagoudas D.C. (2014). Characterization of electrical and thermal properties of carbon nanotube/epoxy composites. Compos. B Eng..

[4] Ibrahim K.S. (2013). Carbon nanotubes-properties and applications: A review. Carbon Lett..

[5] Natsuki T., Natsuki J. (2023). Constitutive modeling of mechanical behaviors of carbon-based CNTs and GSs, and their sensing applications as nanomechanical resonators: A review. Nanomaterials.

[6] Thostenson E.T., Ren Z., Chou T.W. (2001). Advances in the science and technology of carbon nanotubes and their composites: A review. Compos. Sci. Technol..

[7] Zaporotskova I.V., Boroznina N.P., Parkhomenko Y.N., Kozhitov L.V. (2016). Carbon nanotubes: Sensor properties. a review. Mod. Electron. Mater..

[8] Huang X.G., Pang R., Yang M.D., Zhang S.P., Guo F.M., Xu J., Zhang Y.J., Cao A.Y., Shang Y.Y. (2023). Flexible gas sensors based on carbon nanotube hybrid films: A review. Adv. Mater. Technol..

[9] Schroeder V., Savagatrup S., He M., Lin S., Swager T.M. (2019). Carbon nanotube chemical sensors. Chem. Rev..

[10] Zhilyaeva M.A., Asiyanbola O.A., Lomakin M.V., Mironov D.M., Voloskov B.S., Mikladal B., Tsetsereukou D.O., Fedorov F.S., Vershinina A.I., Shandakov S.D. (2022). Tunable force sensor based on carbon nanotube fiber for fine mechanical and acoustic technologies. Nanotechnology.

[11] Paul S.J., Sharma I., Elizabeth I., Gahtori B., Manikandan R.M., Titus S.S., Chandra P., Gupta B.K. (2020). A comparative study of compressible and conductive vertically aligned carbon nanotube forest in different polymer matrixes for high-performance piezoresistive force sensors. ACS Appl. Mater. Interfaces.

[12] Kim J.S., Kim G.W. (2017). Hysteresis compensation of piezoresistive carbon nanotube/ polydimethylsiloxane composite-based force sensors. Sensors.

[13] Li A., Bogdanovich A.E., Bradford P.D. (2015). Aligned carbon nanotube sheet piezoresistive strain sensors. Smart Mater. Struct..

[14] Cullinan M.A., Culpepper M.L. (2010). Carbon nanotubes as piezoresistive microelectromechanical sensors: Theory and experiment. Phys. Rev. B.

[15] Wen L., Nie M., Wang C., Zhao Y.N., Yin K., Sun L. (2022). Multifunctional, light-weight wearable sensor based on 3D porous polyurethane sponge coated with MXene and carbon nanotubes composites. Adv. Mater. Interfaces.

[16] Fei Y.P., Chen F., Fang W., Xu L.X., Ruan S.L., Liu X.H., Zhong M.Q., Kuang T.R. (2020). High-strength, flexible and cycling-stable piezo-resistive polymeric foams derived from thermoplastic polyurethane and multi-wall carbon nanotubes. Compos. B Eng..

[17] Wajahat M., Lee S., Kim J.H., Chang W.S., Pyo J., Cho S.H., Seol S.K. (2018). Flexible strain sensors fabricated by neniscus-guided printing of carbon nanotube−polymer composites. ACS Appl. Mater. Interfaces.

[18] Tang Z., Jia S., Shi S., Wang F., Li B. (2018). Coaxial carbon nanotube/polymer fibers as wearable piezoresistive sensors. Sens. Actuators A.

[19] Hu N., Karube Y., Yan C., Masuda Z., Fukunaga H. (2008). Tunneling effect in a polymer/carbon nanotube nanocomposite strain sensor. Acta Mater..

[20] Natsuki T., Endo M., Takahashi T. (2005). Percolation study of orientated short-fiber composites by a continuum model. Physica A.

[21] Wang J., Musameh M. (2004). Carbon nanotubes screen-printed electrochemical sensors. Analyst.

[22] Chen K., Gao W., Emaminejad S., Kiriya D., Ota H., Nyein H.Y.Y., Takei K., Javey A. (2016). Printed carbon nanotube electronics and sensor systems. Adv. Mater..

[23] Fan Z., Tao X., Dharuman G., Li X.D., Dong L.X. (2016). Modeling and simulation of an ultrasensitive electron tunneling position/force nanosensor. RSC Adv..

[24] Kim D.H., Chang K.J. (2002). Electron transport in telescoping carbon nanotubes. Phys. Rev. B.

[25] Hafner J.H., Cheung C.L., Lieber C.M. (1999). Growth of nanotubes for probe microscopy tips. Nature.

[26] Yan Y., Miao J., Yang Z., Xiao F.X., Yang H.B., Liu B., Yang Y. (2015). Carbon nanotube catalysts: Recent advances in synthesis, characterization and applications. Chem. Soc. Rev..

[27] Eveleens C.A., Irle S., Page A.J. (2019). How does acetonitrile modulate single-walled carbon nanotube diameter during CVD growth?. Carbon.

[28] Moore K.E., Cretu O., Mitome M., Golberg D. (2016). In situ cyclic telescoping of multi-walled carbon nanotubes in a transmission electron microscope. Carbon.

[29] Cumings J., Zettl A. (2000). Low-friction nanoscale linear bearing realized from multiwall carbon nanotubes. Science.

[30] Kang J.W., Byun K.R., Kwon O.K., Choi Y.G., Hwang H.J. (2010). Gigahertz frequency tuner based on a telescoping double-walled carbon nanotube: Molecular dynamics simulations. Mol. Simul..

[31] Ansari R., Motevalli B. (2011). On new aspect of nested carbon nanotubes as Giahertz oscullators. J. Vib. Acoust..

[32] Legoas S.B., Coluci V.R., Braga S.F., Coura P.Z., Dantas S.O., Galvao D.S. (2003). Molecular-dynamics simulations of carbon nanotubes as gigahertz oscillators. Phys. Rev. Lett..

[33] Kang J.W., Lee K.W. (2014). Engineering the resonance frequency of carbon-nanotube oscillators via a telescoping outertube. J. Korean Phys. Soc..

[34] Zhang W., Xi Z.H., Zhang G.M., Li C.Y., Guo D.Z. (2008). Multiple telescoping extension of multiwalled carbon nanotubes and its application in atomic force microscopy. J. Phys. Chem. C.

[35] Jensen K., Girit C., Mickelson W., Zettl A. (2006). Tunable nanoresonators constructed from telescoping nanotubes. Phys. Rev. Lett..

[36] Natsuki T., Ni Q.Q., Elishakoff I. (2013). Influence of the axial compression on the natural frequency of AFM probes using double-walled carbon nanotubes with different wall lengths. Appl. Phys. A.

[37] Cao G.X., Chen X., Kysar J.W. (2005). Strain sensing of carbon nanotubes: Numerical analysis of the vibrational frequency of deformed single-wall carbon nanotubes. Phys. Rev. B.

[38] Nejad M.Z., Hadi A. (2016). Eringen’s non-local elasticity theory for bending analysis of bi-directional functionally graded Euler–Bernoulli nano-beams. Int. J. Eng. Sci..

[39] Abdollahi R., Boroomand B. (2014). Nonlocal elasticity defined by Eringen’s integral model: Introduction of a boundary layer method. Int. J. Solids Struct..

[40] Eringen A.C. (1984). Plane waves in nonlocal micropolar elasticity. Int. J. Eng. Sci..

[41] Heireche H., Tounsi A., Benzair A., Mechab I. (2011). Sound wave propagation in single-walled carbon nanotubes with initial axial stress. J. Appl. Phys..

[42] Kumar D., Heinrich C., Waas A.M. (2008). Buckling analysis of carbon nanotubes modeled using nonlocal continuum theories. J. Appl. Phys..

[43] Zhang Y.Q., Liu G.R., Xie X.Y. (2005). Free transverse vibrations of double-walled carbon nanotubes using a theory of nonlocal elasticity. Phys. Rev. B.

[44] Yoon J., Ru C.Q., Mioduchowski A. (2002). Noncoaxial resonance of an isolated multiwall carbon nanotube. Phys. Rev. B.

[45] Suna C., Liu K. (2009). Dynamic column buckling of multi-walled carbon nanotubes under axial impact load. Solid State Commun..

[46] Enomoto K., Kitakata S., Yasuhara T., Ohtake N., Kuzumaki T., Mitsuda Y. (2006). Measurement of Young’s modulus of carbon nanotubes by nanoprobe manipulation in a transmission electron microscope. Appl. Phys. Lett..

[47] Batra R.C., Gupta S.S. (2008). Wall thickness and radial breathing modes of single-walled carbon nanotubes. J. Appl. Mech..

[48] Lei X.W., Natsuki T., Shi J.X., Ni Q.Q. (2011). Radial breathing vibration of double-walled carbon nanotubes subjected to pressure. Phys. Lett. A.

[49] Yap H.W., Lakes R.S., Carpick R.W. (2007). Mechanical instabilities of individual multiwalled carbon nanotubes under cyclic axial compression. Nano Lett..

[50] Shima H. (2011). Buckling of carbon nanotubes: A state of the art review. Materials.

[51] Akita S., Nishio M., Nakayama Y. (2006). Buckling of multiwall carbon nanotubes under axial compression. Jpn. J. Appl. Phys..

[52] Wang Q., Wang C.M. (2007). The constitutive relation and small scale parameter of nonlocal continuum mechanics for modelling carbon nanotubes. Nanotechnology.

[53] Natsuki T., Matsuyama N., Ni Q.Q. (2015). Vibration analysis of carbon nanotube-based resonator using nonlocal elasticity theory. Appl. Phys. A.

[54] Hosseini-Hashemi S., Nazemnezhad R., Bedroud M. (2014). Surface effects on nonlinear free vibration of functionally graded nanobeams using nonlocal elasticity. Appl. Math. Model.

